# Selective inhibitors of trypanosomal uridylyl transferase RET1 establish druggability of RNA post-transcriptional modifications

**DOI:** 10.1080/15476286.2015.1137422

**Published:** 2016-01-20

**Authors:** Amy Cording, Michael Gormally, Peter J. Bond, Mark Carrington, Shankar Balasubramanian, Eric A. Miska, Beth Thomas

**Affiliations:** a The Gurdon Institute, University of Cambridge, Cambridge, UK; b Department of Chemistry, University of Cambridge, Cambridge, UK; c Cancer Research UK Cambridge Institute, Li Ka Shing Center, Cambridge, UK; d National Center for Advancing Translational Sciences, National Institutes of Health, Bethesda, MD, USA; e Bioinformatics Institute (A*STAR), Singapore; f Department of Biological Sciences, National University of Singapore, Singapore; g Department of Biochemistry, Cambridge, UK

**Keywords:** African trypanosomiasis, drug-discovery, non-coding RNA, post-transcriptional modification, RET1, RNA modifications, TUTase, uridylylation, trypanosome

## Abstract

Non-coding RNAs are crucial regulators for a vast array of cellular processes and have been implicated in human disease. These biological processes represent a hitherto untapped resource in our fight against disease. In this work we identify small molecule inhibitors of a non-coding RNA uridylylation pathway. The TUTase family of enzymes is important for modulating non-coding RNA pathways in both human cancer and pathogen systems. We demonstrate that this new class of drug target can be accessed with traditional drug discovery techniques. Using the *Trypanosoma brucei* TUTase, RET1, we identify TUTase inhibitors and lay the groundwork for the use of this new target class as a therapeutic opportunity for the under-served disease area of African Trypanosomiasis. In a broader sense this work demonstrates the therapeutic potential for targeting RNA post-transcriptional modifications with small molecules in human disease.

## Introduction

RNA plays a vital role in multiple cellular processes, and is involved in many disease states, yet drugs that directly target RNA are rare.^1,2^ Interest in how this rich source of potential drug targets can be can be tapped continues to grow. In humans, mRNA (mRNA) is a minor component of total cellular RNA and accounts for less than 5% of the transcribed genome. Instead, the transcriptome is overwhelmingly populated with a complex mixture of non-coding RNAs (ncRNAs) responsible for a diverse array of regulatory processes. Such ncRNAs are emerging targets in human disease, such as cancer, or for the treatment of parasites, such as the trypanosomes, which rely on unique ncRNA machinery for survival.

One of the principal challenges of targeting a particular RNA with a small molecule drug is the lack of well-defined secondary and tertiary structures. A key requirement for a small molecule ligand to achieve good potency is a well-defined fit in a binding pocket, allowing for multiple interactions to be made between the drug and its target. RNA adopts many transient conformations that are generally short-lived and rarely fold to generate the cavities that are characteristic of the active sites of many druggable enzymes. Thus, a promising strategy to sidestep this difficulty would be to target upstream of the RNA transcript by inhibiting proteins that themselves modify and regulate RNA.

To date, drugs that act either directly on ncRNA or upstream via RNA-modifying enzymes are few in number and unselective. For example 5-azacytidine inhibits the cytosine methylation of both DNA and RNA.^3^ Similarly, enoxacin has been shown to act on TRBP protein to inhibit microRNA (miRNA) biogenesis in addition to its originally identified mode of action, the inhibition of topoisomerase II.^4^ No small molecule therapies specifically targeting non-coding RNAs yet reached the clinic.^5^

One potential class of targets in this field is the RNA terminal uridylyl transferase (TUTase) family, which is comprised of enzymes that modulate the function of target RNA by the sequential addition of uridines to the 3′ terminus.^6^ The effects of these modifications are system dependent, and can mark a substrate for degradation, act as an activation step or prevent downstream processing.^7^ TUTase family enzymes have been shown to act on both coding and non-coding RNA.^7^ Crystal structures of uridylyl transferases reveal a classically druggable active site and make them attractive targets for small molecule therapy.^8^ There are both endogenous and exogenous examples of RNA uridylyl transferases relevant in human disease, for example, human TUT4, which controls the stability of the Let-7 family of miRNAs and has been linked to some forms of cancer.^9,10^ Complementary to this work, the first TUT4 inhibitors have been recently reported in this journal.^11^ The trypanosome TUTase, RET1, has multiple essential roles for the parasite's survival in a host, including the uridylylation of long non-coding guide RNAs (gRNAs).^12^ Infection of human hosts by trypanosomes is responsible for several illnesses including Human African Trypanosomiasis (sleeping sickness) and Chagas disease which cause devastation in the developing world. Current therapies are inadequate and can be fatal.^13^ Previously, RNAi experiments have shown that RET1 expression is essential for *T. brucei* viability. In the absence of RET1, trypanosomes are unable to employ their unique RNA editing mechanism which is dependent on the efficient polyuridylation of gRNA by the enzyme. The resultant failure in RNA regulation results in complete arrest in cell division followed by massive parasitic cell death.^12^

The lifecycle of *T. brucei* is complex with many developmental transitions. Two developmental forms can be grown in culture and are experimentally accessible, the mammalian bloodstream form and the procyclic form from the tsetse fly midgut. The two forms respond differently to chemical treatments. The bloodstream form is clearly of most interest and relevant for the treatment of human disease. Previously published results have demonstrated that RET1 is essential for the viability of *T. brucei* in its procyclic form.^12^ Its importance in the bloodstream form is also supported by data from a whole genome RNAi screen and our own work (see Supplementary Information).^14,15^

RET1 is therefore a particularly relevant target in this disease context. In the present study, we develop a high-throughput assay that identifies inhibitors of uridylylation activity. We apply this methodology in a proof of concept screen using RET1 to demonstrate that TUTases represent a tractable target for drug discovery.

## Results

### Detection of RET1 driven uridylylation of RNA

In order to develop and use an assay suitable for HTS screening for inhibitors of RET1 from *T. brucei* (Kret1, C9ZSY2_Tb927.7.3950), we first required a robust, practical assay system with which we could detect and optimise enzyme activity. Previously reported detection of TUTase activity was achieved by measuring the increased length of RNA as the enzyme adds successive uridines. This was accomplished by radioactive labeling of the nucleotide substrate and quantification of the higher molecular weight products on a denaturing agarose gel.^16,17^ The use of radioactivity in such assays has historically been driven by the need to identify potentially very low quantities of a single substrate from a mixed population of RNA derived from a biological sample. The use of purified RNA *in vitro* overcomes such challenges. Thus, to improve throughput we optimised a non-radioactive, gel-based protocol using Sybr Gold staining for vizualization of RET1 activity at RNA concentrations similar to those used in radioactive experiments.^16,17^ This simple but important development greatly increased the ease with which we could optimise HTS conditions and also provided an orthogonal, gel-based assay to verify hits eventually resulting from our screen.

**Figure 1.**
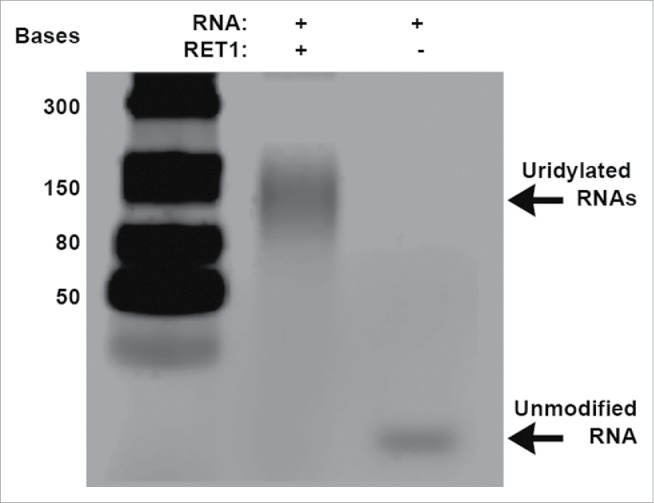
Gel based assay for RET1 activity. RET1 catalyzes the addition of terminal uridines resulting in a poly-U RNA product of approximately 150 bases. In the absence of RET1, the 24 base RNA target remains unmodified.

Full-length RET1 protein (975aa) was expressed in *E. coli* as an N-terminal GST-tagged fusion protein and purified on glutathione sepharose beads as described in the Supplementary Information (gel provided in Fig. S1 and Fig. S2). Starting from the conditions reported by Aphasizheva and Aphasizhev (2010) the uridylylation reaction was optimized to meet our screening requirements of a medium-throughput robust assay and analyzed by gel electrophoresis.^17^ The RNA substrate is elongated from 24 to approximately 150 bases via RET1 catalyzed uridylation (Fig. 1 see *Experimental Section* for detailed assay protocol). We used negative controls to validate this assay, including replacement of RET1 on glutathione sepharose beads with glutathione sepharose beads pre-incubated with lysed *E. coli* cells, beads purified with un-induced *E. coli* extract and a GST tagged N-terminus truncation of RET1 (300aa), which does not contain the enzyme active site.

### Development of a high-throughput assay for inhibitors of RET1 activity

In order to screen a large panel of molecules, we developed a solution based high-throughput assay to monitor the activity of RET1. As RET1 catalyzes the ligation of terminal uridyl groups to the 3′ end of a target ncRNA, the enzyme consumes UTP and produces pyrophosphate (PPi) as a by-product. Thus we quantified the production of PPi to measure RET1 activity over time. While luciferase coupled PPi detection assays of this nature have previously been used to monitor phosphotransferase and other enzyme activities, their use in monitoring the post-translational modification of nucleic acids is recent, as discussed in the contemporary study on TUT4.^11^

**Figure 2.**
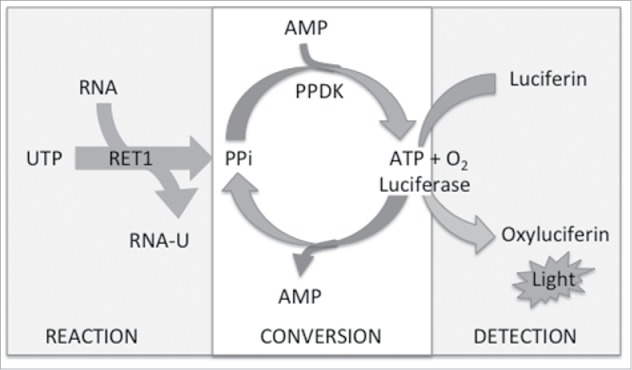
Schematic for the luciferase coupled HTS assay to detect RET1 activity (PPKD – pyruvate phosphate dikinase).

An assay coupling the RET1-catalyzed RNA uridylylation with commercially available PPiLight™ reagents (Lonza) was developed. During the “reaction” step (Fig. 2), poly-U tailing of RNA proceeds with the consumption of UTP and production of PPi. During subsequent “conversion” PPi is used to form ATP by pyruvate phosphate dikinase (PPKD) within the assay medium. Finally, “detection” is facilitated by an ATP-driven luciferase bioluminescent readout. Thus, light output is proportional to PPi concentration and hence RET1 activity. Using the gel-based assay described above, we were able to determine optimal assay conditions compatible with “reaction,” “conversion” and “detection” stages of the reaction and also maintained RET1 activity.

In order to identify substrate-competitive inhibitors, we optimised assay conditions at the K_m_ of both RNA and UTP. At these concentrations there is an optimal window for competitive inhibition to be observed. Measured K_m_ values for RNA substrate (25 nM) and UTP (50 μM), were consistent with those previously reported.^18^

Finally, we miniaturized the assay volume, and simplified the liquid dispense steps to make them amenable to automated dispensing (see *Experimental Section* for detailed assay protocol). As a result, a robust and reliable assay for the detection of RET-1 activity in both 384 and 1536 well format was developed. In order to quantify the statistical power of the high-throughput formats, we calculated the Z’ factor for our assay. A value above 0.5 indicates that more than 12 standard deviations separate the positive and negative controls. In calibration runs for the 384-well format, with heat inactivated protein providing the positive inhibition control, the signal-to-background ratio was approximately 18 and the Z’ factor was 0.77, indicating a reproducible and robust screen.

### Counter-screen to identify false positives

During screening, false positives could potentially arise from inhibition of the conversion or detection steps in this assay. To address this we designed a parallel counter-screen assay in which direct addition of PPi replaced the RET1 reaction step. Compounds that decreased the luminescence signal this assay would indicate inhibition of the “conversion” or “detection” steps rather than direct inhibition of RET1. Any compounds giving rise to a luminescence response of similar magnitude in both the RET1 screen and the PPi counter screen were likely to be acting downstream of RET1. Such compounds were deemed not to be *bona fide* RET-1 inhibitors, and were discarded as false positives.

### Identification of small molecule inhibitors of RET1 uridylylation activity

We used the developed HTS protocol to screen a pilot library of 3,000 compounds with the intention to generalize the use of this assay in the screening of TUTases. Using RET1 we had three aims; first to identify areas of chemical space in which to focus the future search for inhibitors, secondly to investigate the potential for a fragment based screening approach in this area and thirdly to identify the first inhibitors of this class of enzyme, potentially for use as tool compounds for the further investigation of the effects of RET1 inhibition. The compound source was a curated NCGC FDA Pharmacology library of previously approved drugs. This screening deck was chosen because the compounds are well characterized and have been previously used *in vivo*. This is an attractive strategy where access to ADMET assays is limited and where use of compounds with a previously established *in vivo* profile can facilitate and accelerate future cell based or *in vivo* work.

Compounds were initially screened at two concentrations from DMSO stock in 1536 well format. The shortlist of hits was generated by visual inspection of the data based on overall potency and chemical tractability. False positives were identified and discarded using the counter-screen described above. Subsequent to primary screen and counter screening, 30 compounds were selected for follow-up, representing an acceptable hit rate of 1%. These hits were progressed to the next validation step.

### Validation of active compounds

To appropriately identify the inhibitors that function directly on RET1 uridylylation, we screened the 30 short-listed hits in the orthogonal gel-based assay described above. This assay allowed for qualitative verification of the inhibitory activity of the hits from the luciferase assay and classification of compounds as inactive, moderate or strong inhibitors (Table 1).

**Table 1.** Summary of performance of validated RET1 inhibitors. Table provides qualitative activity in gel based assay, inhibition of RET1 in PPi assay 384-well follow-up (reported either as IC_50_ or % inhibition at 1mM), and qualitative determination of UTP competitive binding. *nd* = value not determined for this substrate.

ID	Structure	Gel Assay	RET1 IC_50_
1		Strong	70 µM
2		Strong	2.0 µM
3		strong	500 µM
4		medium	7%
5		weak	5%
6		medium	12%
7		strong	8 µM
8		strong	30%
9		strong	8 µM
10		strong	15 µM
11		strong	5 µM

**Figure 3.**
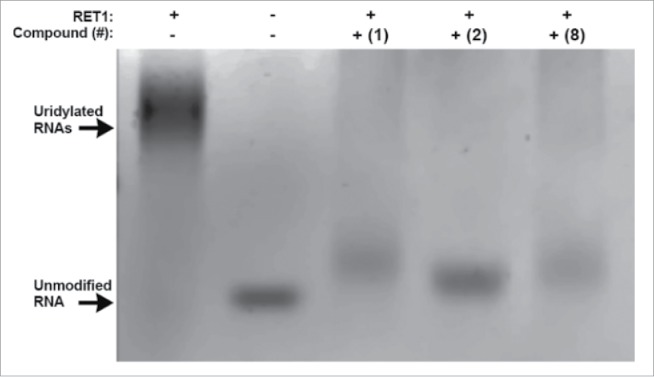
Verification of RET1 inhibitors at 50 µM by gel based assay. Lanes from left to right: Positive control, RET1 is active in assay buffer conditions (5% DMSO) and polyuridylates target RNA producing high molecular weight products; Negative control, in the absence of RET1 RNA is not elongated; Compounds 1, 2, and 8 effectively inhibit the ability of RET1 to uridylate RNA.

Representative gels for compounds **1**, **2** and **8** (Fig. 3), demonstrate a clear reduction in RNA elongation. Of the 30 putative inhibitors, 11 showed moderate or strong activity (see Fig. S3 for complete gels). These 11 compounds were again cycled through the original screening assay, this time in lower-throughput (384-well) format and over a wide dose-range in order to determine the IC_50_s of inhibition. The ranking of IC_50_ values for these compounds was in agreement with their qualitative assessment by gel-based assay giving further confidence in these two complementary assays (Table 1).

As is to be expected from a pilot screen of this size, the potency of these hits is unlikely to be sufficient for *in vivo* exploitation. However, they provide useful insight for curating libraries in future large scale screens or for the rational design of novel inhibitors. Ataciguat (**1**) and exifone (**2**) have both been entered into clinical trials for unrelated indications.^19,20^ Exifone has also been identified as an anti-protozoan in the case of malaria, although a different mode of action is proposed, polypharmacology may contribute to its activity.^21^ Additionally a number of common structural motifs were observed including benzyl thiazolidinediones (compounds **3**, **4** and **5**), a structural class which has given rise to successful drugs such as Rosiglitazone,^22^ and estrogen mimetics (**6** and **7**).^23,24^ Thorough investigation of chemical space around these hit clusters could be fruitful in providing more potent hits. This set of RET1 inhibitors demonstrates that drug-like molecules can indeed target this class of enzymes.

Additionally, we observed some activity in small fragment-like molecules, for example compounds **6** and **8**, despite the fact that fragment-like molecules were poorly represented in this screening deck. With the wealth of structural information in this area derived from a number of TUTase crystal structures, a fragment based approach would be suitable for this class of target.^25^

Although not normally considered drug-like, the aliphatic quaternary amines (**9**, **10**, **11**) are reported here because compound **9** has been reported to have efficacy in the topical treatment of the related pathogen Leishmania.^26^ Of note is the structural similarity of these compounds to miltefosine, which was approved by the FDA for oral treatment of Leishmaniasis. The specific mode of action for miltefosine is unknown (http://www.centerwatch.com/drug-information/fda-approved-drugs/drug/1311/impavido-miltefosine). Homology between RET1 in *T. brucei* and *Leishmania infantum* is high in the domains surrounding the active site (Fig. S8) and it is plausible that the RET1 inhibition that we report here may indeed contribute to their mode of action.

### Hit compounds are toxic to trypanosomes in culture

As ataciguat (**1**) and exifone (**2**) were among the most potent drug-like hits, and have been characterized previously in human subjects, we performed preliminary experiments to evaluate their effect on the trypanosome *T. brucei*. The lifecycle of *T. brucei* is split into distinct phases depending on whether the host is an insect or mammalian. The organism can respond differently to chemical treatments in the different forms and the blood form is clearly the most relevant for the treatment of human disease. We found that compounds **1** and **2** are toxic to both blood and insect forms at concentrations close to their IC50 values (70μM and 2μM respectively) (Fig. S4). This is an important characteristic of these hit compounds, and is consistent with a RET1 driven mode of action. In the case of exifone, its potency is sufficient to rule out non-specific cytotoxicity, since it is not toxic to PC12 cells at concentrations of 10 μM.^27^ A larger scale screen will lead to more potent inhibitors for use as tool compounds or hits for a drug discovery program in this area. The chemical space identified in this pilot study can be exploited in such future screening efforts.

### Homology model predicts binding modes of ataciguat (1) and exifone (2)

Several crystal structures of uridylyl transferases have been published in recent years. However to date, attempts to crystallize RET1 have been unsuccessful due to its preference to form complex interactions with other proteins. Using the wealth of information derived from crystal structures of other TUTases, a homology model for RET1 was generated.

We performed manual alignment of RET1 with its close homolog Tb RET2 (pdb 2B51),^28^ followed by a homology modeling and energy-scoring protocol. The best-scoring models exhibited excellent overlay of all 20 key ligand binding and catalytic motif residues in comparison to the Tb RET2 template. Subsequent pair-wise clustering of the model ensemble based on these residues revealed limited rotameric variability among cluster representatives, confirming the quality of the models. The six clusters revealed a limited root-mean square deviation (RMSD) distribution of just ˜1Å, and the central models from the two most populated clusters (covering >95 % of all models) was chosen for subsequent analysis and docking. Comparison with alternative models generated from the crystal structure of the apo and bound states of the less closely related TbTUT4 (pdb 2IKF) revealed RMSD distributions of ˜2-4 Å, confirming TbRET2 as the better starting point. Full details of the generation of the homology models and docking protocol can be found in in the Supplementary Information.

**Figure 4.**
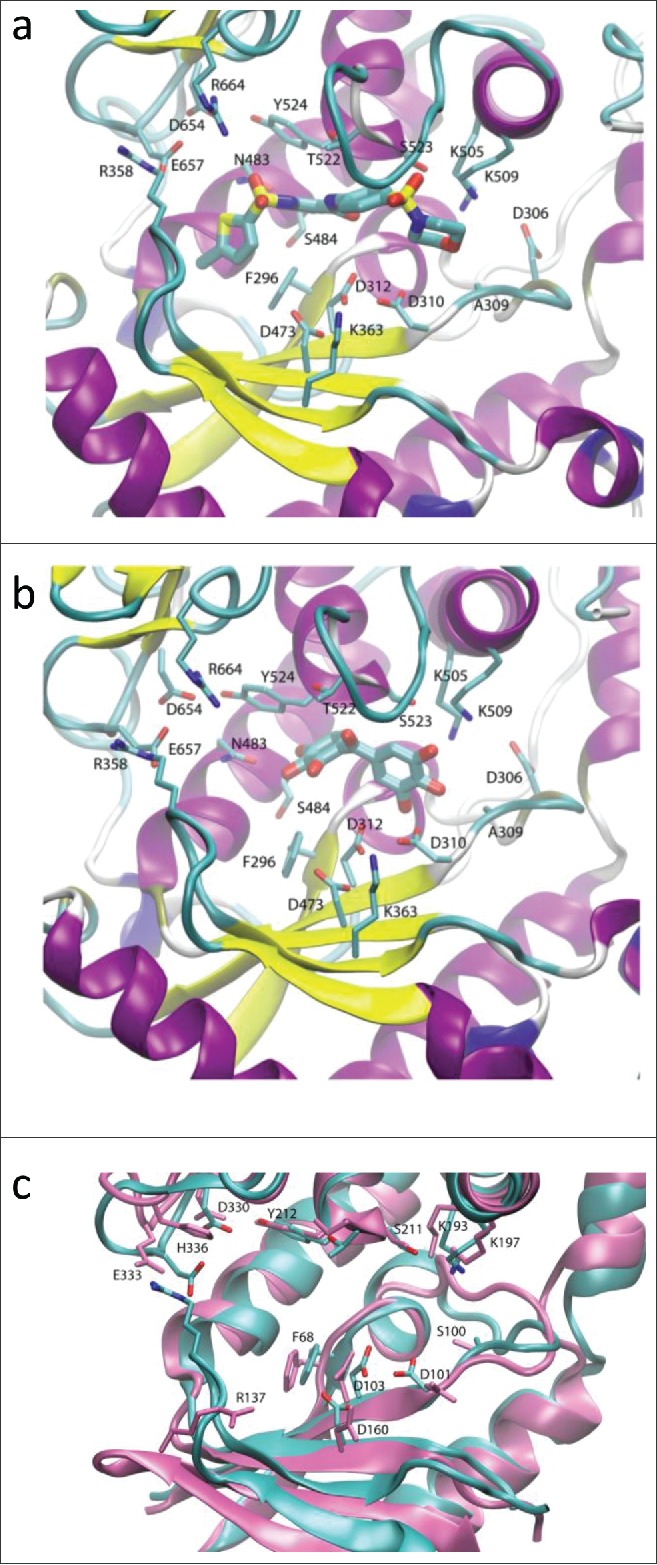
Putative binding modes predicted using the RET1 homology model. a) Ataciguat (**1**) and b) Exifone **(2)** docked in the RET1 apo binding site showing the most populated cluster. In a) and b), the protein fold is shown in cartoons representation, colored by secondary structure (β-sheet in yellow, α-helix in purple, loops in cyan). The extensive N-terminal domain β-loop containing the Arg358-Glu657 salt bridge (labeled) helps to create a snug binding site for inhibitor. c) Overlay of RET1 homology model (cyan, with sidechains in CPK format) and CID1 crystal structure (all mauve). The residues of CID1 crystal structure are labeled, while those of the RET1 homology model are unlabelled but are shown in the same orientation as in a) and b). The presence of His336 in CID1 increases the electrostatic potential at the top of the binding site, leading to separation of Glu333 and Arg137 (residues corresponding to those that form the Arg358-Glu657 salt bridge in RET1) leading to a more open binding site in CID1.

We observed two putative binding modes for ataciguat (**1**). In RET1, both binding modes enable similar interactions at either end of the molecule. In configuration 1 (Fig. 4A), the terminal chlorine group is in a small hydrophobic pocket, Phe296 stacks against the thiophene ring, Tyr524 could potentially stack with the central chlorinated ring and the connected sulfonyl oxygen atoms would form direct or water mediated H-bonds to nearby charged residues Glu657, Arg664, Asp654. These are therefore mostly replacing interactions that would normally be stabilizing the uracil base. Meanwhile, the central carbonyl may interact via water or ionic interactions with the catalytic triad aspartate Asp310/Asp312 carboxylates, with the sulfonyl oxygen atoms next to the morpholine group interacting with Ser523, and the morpholine ring oxygen with Lys509, thus adopting interactions normally reserved for the phosphates. In configuration 2 (Fig. S5), ataciguat (**1**) is rotated by 180 degrees and this pattern is reversed; the morpholine group lies in the small hydrophobic pocket and could stack with Phe296, the central benzene could stack with Tyr524, the intervening sulfonyl oxygen atoms form direct or water mediated H-bonds with Glu657/Arg664/Asp654. The central carbonyl oxygen may form H-bonds with nearby catalytic triad aspartates Asp473, Asp310, Asp312, and sulfonyl oxygen atoms adjacent to the thiophene ring would interact with Lys505/Lys509/Ser523. Either binding mode predicts a similar interaction, leading to a snug fit of the molecule into the elongated RET1 cavity.^29^ The principal reason for this is the fact that the binding site is closed off by the conformation of an N-terminal domain β-loop which protrudes into the active site, stabilized by a salt bridge between Arg358 and Glu657.

Modeling of exifone (**2**) also gave rise to two potential binding modes. In configuration 1, exifone is near to the phosphate-binding end of the site (Fig. 4B). Ring hydroxyls orient toward Lys509/Ser523 and Asp310/Asp312 which would form direct or water-mediated hydrogen bonds with the phosphate coordination/catalytic sites. Further along the binding site interactions are limited other than a ring hydroxyl H-bond with the sidechain of Asp483. In configuration 2 (Fig. S6), exifone is nearer to the base-binding site. Some of the previous interactions are lost, though ring hydroxyls are still near to the catalytic Asp312 carboxylate. In this situation ring hydroxyls are near to base-interaction sites and form direct or water-mediated H-bonds to Asp654 and Glu657, the base stacks with Tyr524 and the central carbonyl may H-bond to Thr522.

### Selectivity for RET1 inhibitors over the homolog CID1

**Figure 5.**
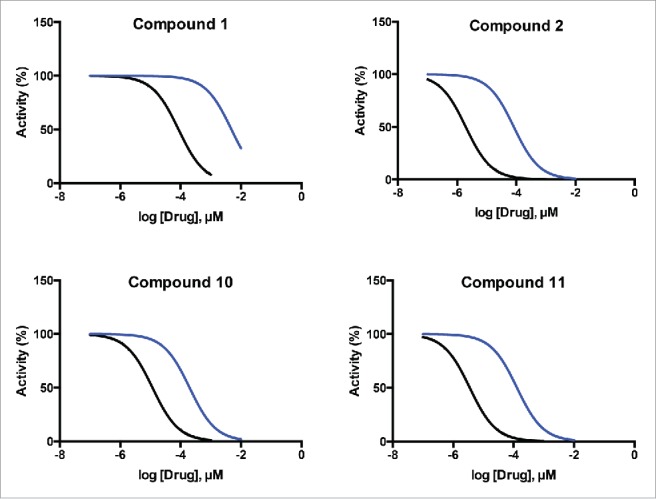
Representative curves showing RET1 selectivity of short-listed hit compounds. Compounds exhibited varying degrees of selectivity for inhibiton of RET1 (black curves) over the yeast TUTase CID1 (blue curves).

One question when investigating a new class of target is whether it is possible to obtain sufficient selectivity over related enzymes to achieve a suitable therapeutic window. Here we report the first evidence for selectivity of inhibitors between members of the TUTase family of enzymes. We adapted our luciferase assay to monitor the activity of the commercially available yeast TUTase CID1 (see Online Methods in Supplementary Information). Again focusing on the drug-like hit compounds that gave complete RET1 inhibition curves, we assessed each for selective inhibition of RET1 over CID1. Representative curves for compounds **1**, **2**, **10**, and **11** are shown (Fig. 5); and IC_50_ values, and calculated fold-selectivity for all compounds tested is provided (Table 2).

**Table 2.** Results from RET1 selectivity experiments performed with top validated hit compounds demonstrate the specific inhibition of the trypanosomal enzyme RET1 over an equivalent yeast TUTase CID1.

	RET1 Inhibition			CID1 Inhibition			
Compound	IC_50_(µM)		SD	IC_50_(µM)		SD	RET1 Selectivity (fold)
1	73	±	12	4800	±	1700	66
2	2.0	±	0.1	88	±	19	43
3	500	±	50	1000	±	100	2
7	8.3	±	0.7	68	±	1.6	8
10	15	±	3.1	270	±	170	17
11	4.5	±	0.8	110	±	56	24

Finally, we used the existing crystal structure of CID1 (pdb 4E80) to build hypotheses for the observed selective inhibition of RET1 over CID1.^30^ CID1 residues are largely conserved when compared with RET1, and in principle would provide similar interactions for compounds such as ataciguat (**1**). However, the closed NTD β-loop conformation of RET1, which as described above leads to a snug fit of inhibitor within its binding cavity, is not present in CID1 despite the conservation of the equivalent salt bridge residues (Arg137 and E333 in CID1). This is demonstrated in Fig. 4C, with residues of CID1 labeled and shown in mauve, overlaid on the RET1 structure shown in cyan in the same orientation as Figs. 4A–B. The salt bridge observed in RET1 is not formed in CID1, as the guanidinium group of Arg137 in CID1 (Arg358 in RET1) is shifted downwards by >10 Å, and the carboxylate of Glu333 in CID1 (Glu657 in RET1) is shifted out of the binding site by 2Å. The loss of the closed binding site would explain the selectivity of Ataciguat for RET1 over CID1. There is a histidine residue, His336, in CID1 which is absent in RET1 but conserved in mammalian orthologues including all known human TUTases (see Fig. S7 for docking model).^30^ This residue increases the local positive electrostatic potential near to the equivalent site where the salt bridge in RET1 is present. We postulate that this increased positive electrostatic potential creates an alternative “binding site” for Glu333 in CID1, and/or leads to repulsion of Arg137, and is thus responsible for destabilization of an otherwise possible Arg137-Glu333 salt bridge (Arg358-Glu657 in RET1), causing loss of the closed binding site, and giving rise to the observed selectivity of the inhibitors for RET1 over CID1 (Fig. 4C).

## Conclusions

Non-coding RNAs remain a little-explored area in drug discovery. The enzymatic machinery responsible for the post-translational modification and control of ncRNAs presents an enticing hub to exploit post translational modifications as drug targets. The TUTase family of enzymes is an integral part of multiple non-coding RNA regulatory pathways. In this study we have demonstrated the suitability of this class of enzymes as a druggable target. Using RET1 as an example we have developed a tractable route for the identification of tool compounds and drug-like inhibitors of TUTases, demonstrating one of the first effective and achievable means of manipulating disease-relevant regulatory RNAs therapeutically.

Employing the screening protocol reported in this study, we identified the first inhibitors of RET1 from a library of 3,000 small molecules. Hit compounds included drug- and fragment-like molecules. We were also able to demonstrate cross-species selectivity between TUTase homologues. Using a homology model of RET1, putative binding modes were solved for the primary hit compounds. Importantly, this model was in agreement with our experimental results and rationalized the selectivity we observed over a related RNA processing enzyme. These results will help to inform further investigations to identify inhibitors with improved potency against RET1 that have the potential to provide a much needed new treatment for African trypanosomiasis.

## Experimental section

### Gel based assay

Individual reactions were made up in 0.2 ml PCR tubes (Peqlab 820264-A). RET1 enzyme (see SI text) was added (0.05 μl, 0.2 mg ml^−1^), UTP (Thermo Scientific R0471 100 mM) was added to a final concentration of 50 μM. RNA substrate (see SI text) was added to a final concentration of 200 nM. Inhibitors were added as required (0.5 μl in DMSO) to a final concentration of 50 μM. When no inhibitor was included 5 % (vol/vol) DMSO was added as control. The volume was made up to 10 μl in buffer D (Tris pH 7.5 10 mM, KCl 200 mM, DTT 1 mM, EDTA 0.5 mM MgCl_2_ 3.2 mM) and the reaction was incubated for 20 mins at 27°C, then inactivated at 65°C for 10 mins. RNA loading dye (10 μl, NEB B363A) was added and tubes were heated (5 mins at 70°C), 10 μl was loaded onto 15 % TBE urea pre-cast gel (Invitrogen EC68852) and run in 1 x TBE buffer for 1.5 h at 130 V. Gels were incubated with Sybr Gold nucleic acid stain (Life Technologies S11494) for 20 mins, then imaged on an ultraviolet trans-illuminator (BioDoc-It imaging system, UVP). Experiments were carried out in duplicate for each inhibitor.

### Luciferase assay 384 well format

Reactions were made up in in Corning® 384 Well Low Flange White Flat Bottom Polystyrene TC-Treated Micro plates (Product #3570). RET1 enzyme was added at 0.1 μl (0.2 mgml^−1^), UTP (Thermo Scientific R0471 100 mM) and RNA substrate final concentrations were equal to their K_m_: 50 μM and 20 nM respectively.^18^ The volume was made up to 10 μl with buffer 6 (Tris pH 7.5 10 mM, KCl 10 mM, DTT 1 mM, MgCl_2_ 3.2 mM) and the reaction was incubated 20 mins at 25°C, then heat inactivated at 65°C for 10 mins. Test compounds were added in 0.5 μl of DMSO stock solution. The positive control included of 0.5 μl of DMSO. After a 10 min cooling period, 5 μl of each of detection and conversion reagents from PPiLight™ Inorganic Pyrophosphate Assay Kit (Lonza, LT07-610) were added. Luminescence was read with a Pherastar Plus micro plate reader. IC_50_ determinations were carried out in triplicate using the luciferase assay protocol in a dilution series of 1 mM to 99 nM.

### Luciferase assay 1536 well format

Minor modifications were made to the large-format assay to adapt the protocol into high-throughput, 1536-well format. Dispense steps were simplified into three steps. First, RET1 (3 µl, 5 µgml^−1^) in buffer 6 was dispensed into each well using a Bioraptr reagent dispenser (Beckman-Coulter). Columns 1 and 4 were reserved for negative controls and received buffer only. Second, the compound library and DMSO controls were dispensed at indicated concentrations in 23 nL using a Kalypsys 1536-pin tool (Wako). Finally, RNA substrate (100 nM), UTP (200 µM) and PPi light reagents were dispensed in 1µL on the Bioraptr. The plates were read for luminescence 20 mins after dispensing using a ViewLux high-throughput charge-coupled device (CCD) imager (Perkin-Elmer). Following this initial read, the reaction was spiked with PPi (1 µL, final concentration 25 nM). The plate was read again in the same fashion to identify no responding wells, which represented off target molecules that inhibited the assay.

## Supplementary Material

Supplemental_Figures_and_Information.docx
