# Supplementary material for: Selective inhibitors of trypanosomal uridylyl transferase RET1 establish druggability of RNA post-transcriptional modifications
Source: RNA Biol. 2016 Jan 20;14(5):611–9. doi: 10.1080/15476286.2015.1137422 (PMC5449093; doi:10.1080/15476286.2015.1137422)
Supplement: Supplemental_Figures_and_Information.docx [file krnb-14-05-1137422-s001.docx]

**SUPPLEMENTARY INFORMATION**

**Selective inhibitors of trypanosomal uridylyl transferase RET1 establish druggability of RNA post-transcriptional modifications**

**Amy Cording^1^, Michael Gormally^2,3,4^, Peter Bond^5,6^, Mark Carrington^7^, Shankar Balasubramanian^2,3^, Eric A. Miska*^1^, Beth Thomas*^2^**

^1^ The Gurdon Institute, University of Cambridge, Tennis Court Road, Cambridge CB2 1QN, UK

^2^Department of Chemistry, University of Cambridge, Lensfield Road, Cambridge, CB2 1EW, UK

^3^ Cancer Research UK Cambridge Institute, Li Ka Shing Centre, Robinson Way, Cambridge, CB2 0RE, UK

^4^ National Center for Advancing Translational Sciences, National Institutes of Health, Bethesda, MD 20892

^5^ Bioinformatics Institute (A*STAR), 30 Biopolis Str, Singapore 138671

^6^ Department of Biological Sciences, National University of Singapore, 14 Science Drive 4, 117543 Singapore

^7^ Department of Biochemistry, Tennis Court Road, Cambridge CB2 1QW

Correspondence should be addressed to BT at [bthomas@ccdc.cam.ac.uk](mailto:bthomas@ccdc.cam.ac.uk) and EAM at [eam29@cam.ac.uk](mailto:eam29@cam.ac.uk)

**RET1 is a viable target for the treatment of human infections of *T. brucei***

The lifecycle of *T. brucei*  is complex with many developmental transitions. Two developmental forms can be grown in culture and are experimentally accessible, the mammalian bloodstream form and the procyclic form from the tsetse fly midgut. The two forms respond differently to chemical treatments. The bloodstream form is clearly of most interest and relevant for the treatment of human disease.  Previously published results have demonstrated that RET1 is essential for the viability of *T. brucei* in its procyclic form. (11) Its importance in the bloodstream form is also supported by data from a whole genome RNAi screen. (13) (14) We have repeated and extended this work to demonstrate that RNAi induced depletion of RET1 decreases growth rate in both the procyclic insect and bloodstream forms of this parasite.

A tetracycline-inducible RET1 RNAi construct was made as described in the supplementary information, and stable cell lines were generated by clonal selection. After RNAi induction the depletion of RET1 was verified by western blot using an anti-RET1 antibody (donated by R. Aphasizhev) shown in Figure 1. Trypanosome proliferation was measured over a time course of several days after induction of RNAi and it was found that RET1 depletion caused a reduction in trypanosome proliferation at around 72 hours post induction in the bloodstream form (Fig. 1). This is consistent with accumulated damage caused by malfunctioning RNA editing and is in line with the findings of Aphasizheva for the procyclic form. These data support the choice of RET1 as a viable target for the treatment of human infections of *T. brucei.*

**A**

Loading control


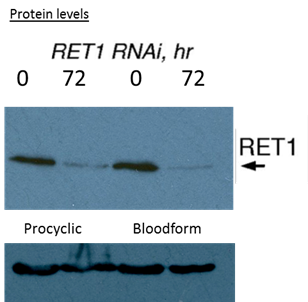

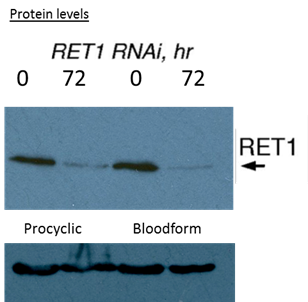


Procyclic Bloodform

RET1

RET1 RNAi, hrs

0 72 0 72

**B**

**Figure 1** RNAi induced depletion of RET1 causes cell arrest. (A) Western blot analysis of RET1 knockdown by inducible RNAi using *anti*-RET1 antibody in the procyclic and blood form shows decreased levels of RET1 protein present at 72h post-induction. Loading control was β-Tubulin. (B) Cumulative growth curve of cell cultures with (blue) and without (red) RNAi induction. Cells were induced at 5 × 10^5^/ml and diluted every 24 h to the same density.

**Online Methods**

**Cloning and expression of N-term GST tagged trypanome RET1 protein for expression in *E.coli***

The N-terminal GST tagged RET1 *E.coli* expression construct was made via PCR amplification of the entire coding region of the RET1 gene (1-2928 bp), using primers M5697, M5698. The PCR reaction was set up as follows: 60ng of genomic DNA, 0.2uM final concentration of each primer, 1x Phusion Buffer, 0.2mM final concentration of dNTP, 0.5 ul Phusion High fidelity DNA polymerase (NEB M05305) and made up to a total volume of 50 ul. PCR program was run: 1 cycle 98 °C 30 s, followed by 30 cycles of 98 °C 10 s, 57 °C 30 s, 72 °C 60 s, then 1 cycle at 72°C for 10 min. The entire PCR reaction was then mixed with DNA loading dye and run on a 1% agarose gel. The PCR amplified region was identified by size against Bioline Hyperladder 1 kb (BIO-33053), cut out and purified with QIAquick gel extraction kit (Qiagen 28704). It was then sequenced (Beckman coulter genomics service) and was then sub-cloned into the gateway entry vector pDONR221 (Invitrogen 12536-017) using standard BP Clonase kit (Invitrogen 11789-013) and reaction procedure. Clones were then transformed, plated and selected for with antibiotic kanamycin resistance. Surviving clones were mini prepped (QIAgen 27104). Positive clones were then recombined with *E.coli* N-term GST tagged pGEX-2TK expression vector (GE healthcare 28-9546-46) which had been previously modified to include a GATEWAY destination cassette inserted into its SmaI site using standard LR Clonase kit (Invitrogen 11791043) and reaction procedure. Clones were then transformed selected for with ampicillin resistance and mini prepped (QIAgen 27104) to give construct 1.

Truncated versions of the RET1 gene were made in exactly the same way as stated above, gene regions started at the same start ATG site and shared forward PCR primer M5697. Truncations were made for the following fragments: 1650bp (550 aa) reverse primer M5872, 900bp (300 aa) reverse primer M5870.

**Expression and purification of N-term GST tagged RET1 protein in *E. coli***

The N-term RET-1 containing GST tagged pGEX-2TK DNA construct was transformed into *E.coli* expression line BL21 (DE3) (Bioline 850-32) and a 250 ml LB broth starter culture was inoculated to grow over night at 37 °C, this was then used to inoculate 10x 1L flasks of LB, which were grown at 37 °C for 1.5 h. 1 ml of 1 M isopropyl-beta-D-thiogalactopyranoside was then added to each flask before incubation at 25 °C for a further 5 h. *E.coli* were collected in 1 L batches by centrifugation at 5,000g for 20 min (Sorvall RC 3B Plus centrifuge). The supernatant was discarded and pellets was re-suspended in 1 x PBS, 0.01 % triton, pH 7.5, DTT 1 mM, 15 ml total volume was used for each pellet. Proteinase inhibitor (1 Roche EDTA free tablet 04693132001 pre dissolved into 50 ml 1 x PBS) and was added at further dilution of 1/50. Samples were then sonicated with Sonics Vibracell, in combination with Jencons PLS stepped micro tip standing sonicator at an amplitude of 25 %, for 10x 5 sec bursts. All of the above steps were carried out on ice. Samples were then centrifuged at 10,000 g for 30 mins at 4 °C. Supernatant was collected in 30ml batches and mixed with 500 ul of (pre-washed in 1x PBS 0.01 % Triton pH 7.5) glutathione sepharose beads (GE healthcare 17-0756-01) and incubated at 4 °C with constant rocking for a further 2 h. Beads were then washed 3 x 5 mins with 10 ml wash buffer (1 x PBS 0.01 % Triton, 400 mM NaCl, MgCl_2_ 3.2 mM) then 3 x washes with buffer D (Tris pH 7.5 10 mM, KCL 200 mM, DTT 1 mM, EDTA 0.5 mM MgCl_2_ 3.2 mM). Beads were then frozen in 50 ul aliquots at -80°C.

For each of the two RET1 truncations, 1L of protein was grown and purified (as above) and reagents were scaled down accordingly.

**SDS page Protein gel and Western blotting for N-Term GST tagged RET1 protein**

6 µl of 0.1-0.2 µg RET1 Protein was mixed with 4µl of NUPAGE 4x sample buffer (Invitrogen NP0007) and run on a 10% Tris-glycine polyacrylamide gel, in 10% SDS Tris-glycine running buffer, with Biorad gel tank system at 130 V for 1.5 hrs with Protein size marker (NEB P7711S). Proteins were then either then stained with coomassie blue for 2hrs, before destaining in standard de stain buffer or proteins were transferred to hyband-ECL membrane (GE healthcare RPN303D) for 1 hr at 4 °C, 100 V for western blot analysis.

For western blot, Membrane washed in 1x TBST (50 mM Tris-Cl, pH7.5, 150mM NaCl, 0.01% Tween) buffer before blocking in 1xTBST 5% dried milk powder for 1hr. After this time Primary mouse monoclonal IgG1 anti GST antibody put on ON at 4 °C, diluted 1:1000 1x TBST 5% dried milk powder (Santa Cruz Biotech 200ug/ml SC-138). The next day membrane washed at RT 3x 20 min each wash in 1x TBST, then secondary antibody was put on for two hrs., anti-mouse IgG horseradish peroxidase (sheep) linked, diluted 1:5000 (GE Healthcare NA931V). After this time, membrane was blotted dry and put on Saran wrap with 1ml of chemiluminescent HRP substrate developer (Millipore WBKLS0500) applied for 5 mins. Membrane was then blotted to remove excess liquid and then put into cassette for imaging, with super RX Fuji medical X-ray film (4741019236). Exposure times of 5 sec-2mins were used. Films processed on Xograph imaging system compact x4 film processor.

Although anti GST staining of the *E.coli* extract showed largely intact protein (figure S1) significant fragmentation on purification was observed (figure S2). Efforts to optimize this procedure in order to reduce fragmentation or further purify RET1 were not successful. The size of the major fragment was between 50 and 60 kDa. Mass spectral analysis of this band from the gel confirmed its size and identity as containing the first 483 aa of the construct. This does not contain the RET1 catalytic region. Constructs 2 (550 aa) and 3 (300 aa) mentioned above were designed and tested to verify the inactivity of these truncated fragments. Constructs 2 and 3 were indeed found to be inactive and we concluded that the presence of such truncations would not generate false positive results. The concentration of active protein was calculated based on the intensity of the observed intact band.

**Cloning of RET1 RNAi construct and creation of stable cell lines in *T.brucei***

RET1 RNAi trypanosome construct was made by PCR amplification of gene region 444 to 1025 bp (primers used M6260,M6261). Both PCR phusion reaction and gel extraction were carried out in the same way as described above. Cloning RET1 444-1025 gene fragment into the vector p2T7-177 was performed by classical cloning cutting both PCR product and vector with BamHI and HindIII and ligating with T4 ligase (NEB M0202) standard procedure. Clones were then transformed and mini prepped (QIAgen 27104) to produce DNA. The p2T7-177 vector with RET1 inserted was then further linearized with NotI before 10ug was co-transfected by electroporation into both procyclic (Lister 427) and bloodstream (Lister 927) *T.brucei* cells with either SmOXp427 or SmOXp927 (S.K Poon et al. Open Biology 2012) which had also been pre linearized with HindIII. For electroporation cells lines were both grown to mid log phase before being re concentrated to 2-3x107. Positive cells lines were then selected for by looking for clones with both phleomycin (2 µg ml^−1^) and puromycin (1 µg ml^−1^) resistance, cell lines were then made and frozen at -80°C.

**Culturing *T.brucei* and RET1 RNAi induction**

Cells were then sub-cultured every 24 h to the same cell density (5 × 10^6^ or 5 × 10^5^ cells ml^−1^ for procyclic and bloodstream form, respectively**.** Procyclic form were grown at 27 °C in SDM-79 medium with 7.5 mg hypoxanthine in 50 mM NaOH (added at 3 ml per L of medium), 10 % FCS, puromycin 1 µg ml^−1^, zeocin 2 µg ml^−1^ and Bloodform grown at 37 °C in HMI-9 medium, 1% pen/strep, 10 % FCS, Puromycin 0.1 µg ml^−1^, zeocin 1 µg ml^−1^.

Cells were set up at “time zero” and antibiotics were removed, after 24 h, RNAi was induced by the addition of 1 µg ml^−1^ doxycycline to the culture medium. A non-induced control was grown side by side for both the procyclic and blood form. Cell density measurements were performed every 24hrs using a hem cytometer.

**Verification of RET1 protein knockdown via western blot**

For procycic form, 5x 10^7^ cells were collected from induced and a non-induced control line, they were pelleted, washed in SDM-79 medium minus FCS, supernatant was then removed and pellet flash frozen, stored at -80 °C. For blood form, 1x10^8^ cells were collected, again from both conditions, washed in HMI-9 medium minus FCS, supernatant was removed and pellet flash frozen, stored at -80 °C.

For both forms, 5x10^6^ cells were resuspended in 1 x PBS, 0.01 % triton, pH 7.5, DTT 1 mM with proteinase inhibitor added (as before). This was mixed with NuPAGE 4x sample buffer (Invitrogen NP0007), heated for 5 mins at 98 °C, before being centrifuged for 10 mins and sample was loaded on gel. Gel was run as before and western blots were performed as described above. For RET1 detection an anti RET1 mouse ascites antibody was used at 1:200 dilution (antibody kindly gifted to use by Ruslan Afasizhev) To equalize samples on the western a monoclonal mouse anti tubulin antibody was used as a loading control (clone DM1A (1:10,000 Sigma-Aldrich). A secondary, horseradish peroxidase linked anti-mouse IgG (sheep), diluted 1:5000 (GE Healthcare NA931V), was used to detect both.

**Treating *T. brucei* cultures with chemical compound candidates**

Wild type blood form strain of trypanosomes were cultured to densities of 5 × 10^5^ cells ml^−1^ at 37 °C in HMI-9 medium, 1 % pen/strep, 10 % FCS, Puromycin 0.1 ug ml^−1^ , zeocin 1 µg ml^−1^. Test compounds in DMSO were added at varying concentrations (DMSO final concentration 1 %). The trypanosomes where then split 1/10 dilution and sub-cultured every 24 h to the same cell density (5 × 10^5^ cells ml^−1^). At 24 hour intervals, cell density measurements were performed using a hem cytometer. Test concentrations were determined by an initial dose finding experiment and compounds were tested at 0.2, 0.5 and 1 times a fatal dose. This was in the region of the IC_50_ for both compounds.

**CID1 poly (U) polymerase**

Poly (U) polymerase from *Schizosaccharomyces pombe*, was obtained from NEB (M0337S).

**Primers for cloning**

M5697 GGGGACAAGTTTGTACAAAAAAGCAGGCTCA ATG GTA AGT AAG TAC CACCGCTTG

M5698 GGGGACCACTTTGTACAAGAAAGCTGGGTA **TCA** ACG AGG GCT CTT GAA TGG AG

M5870 GGGGACCACTTTGTACAAGAAAGCTGGGTA**TCA**CGCCACAGAGCTTCCAAATA

M5872 GGGGACCACTTTGTACAAGAAAGCTGGGTA**TCA**TGATTCAGGAATTTCAAGAG

M6260 CATTCAGGATCCTGAGAGTGATGGGAATTTGGAC

M6261 GTCGCTAAGCTTGGCGTAGTAGCTCTGTGATGGA

**Protein Sequences**

**KRET1 protein sequence 975aa**

MVSKYHRLLQ QGLREEEGVT ERHMVAGGEQ RHGHVDDDNA EGDADFYDQK DERRAKMSNP

KHESANVSAG GKQNRSVRDC LPGSLPPVAN TSTDAAVRFD RERKNAGHGI DISCVEGDGA

QMGTYVSTGR SDAKAGGGSS AIGVTADDES DGNLDTDGSD ASEGDEVEST TDADVYGEDD

TTEGPRGGVR LYSCDACPHA VFTTHAALLA HAEEHHADLL PDHARLRRIA QKLNPVWNRA

LNARRNTITS WGKKIFHVAA QRDAGESKMQ EAHRARAQLE CVVRRWHDKA RVFIFGSSVA

MGVWDGTADI DFAVVDVDAM ERGSWPPLEK NAVRSITELL RRVGFSFVNL EPISHARVPI

IKHHASSPIL TVARRDAEDV VARSIRFILN GPATREDRLL LEGSVRDAVG PTGVQQVWWN

RTSDMMSATL ESTTAAVRAA MCSPALASAS LRTKVQPAHD ECRPELYNID FDLSFRAFGI

RNSTLLRKYL LSHPCARPGA IVLKDWSKTS GVNNSVNGYF TSYAINIMWI YYLVQKGYVP

YVDPLEIPES LVNYTDFDPR YTPMIDPEIT NTEREELYKA AGDMLVGFFY FYSFEFDWGH

NVISLNRPGI TTKRMLGWHV EDVVPVASTS VSSGGGGSNV KRHPTRYELC IEDPYEENLN

LGRHIGVTKS LRVRTELYRG LLSLLKEGET RSCVFAAADS SGTPAAGGKQ SAALPARALF

KLMALTTQAI SESRRLPQSN SDNSGRIGNG DNESLTEVGG GHRVEGAGVD PASCAGASLS

SFGEPPIGVH EKTLESIFVE KAPMEFQLVR KVWNWHQLIH RLGYKIHRGH VMPRREVGVR

CTARRDAEET TTELASDVDT TKSLRPGRGL TDTMLRDLSR GYMTLTPEWV AWSAPWVSQH

LRGYSRLTTV RSAVADETPP ALATVPSVVK PPTGEAVMGA MRTTRRNAAP ARRVELLKLW

LWRGISKVTP FKSPR

**550 aa truncation**

MVSKYHRLLQ QGLREEEGVT ERHMVAGGEQ RHGHVDDDNA EGDADFYDQK DERRAKMSNP

KHESANVSAG GKQNRSVRDC LPGSLPPVAN TSTDAAVRFD RERKNAGHGI DISCVEGDGA

QMGTYVSTGR SDAKAGGGSS AIGVTADDES DGNLDTDGSD ASEGDEVEST TDADVYGEDD

TTEGPRGGVR LYSCDACPHA VFTTHAALLA HAEEHHADLL PDHARLRRIA QKLNPVWNRA

LNARRNTITS WGKKIFHVAA QRDAGESKMQ EAHRARAQLE CVVRRWHDKA RVFIFGSSVA

MGVWDGTADI DFAVVDVDAM ERGSWPPLEK NAVRSITELL RRVGFSFVNL EPISHARVPI

IKHHASSPIL TVARRDAEDV VARSIRFILN GPATREDRLL LEGSVRDAVG PTGVQQVWWN

RTSDMMSATL ESTTAAVRAA MCSPALASAS LRTKVQPAHD ECRPELYNID FDLSFRAFGI

RNSTLLRKYL LSHPCARPGA IVLKDWSKTS GVNNSVNGYF TSYAINIMWI YYLVQKGYVP

YVDPLEIPES

**300aa truncation**

MVSKYHRLLQ QGLREEEGVT ERHMVAGGEQ RHGHVDDDNA EGDADFYDQK DERRAKMSNP

KHESANVSAG GKQNRSVRDC LPGSLPPVAN TSTDAAVRFD RERKNAGHGI DISCVEGDGA

QMGTYVSTGR SDAKAGGGSS AIGVTADDES DGNLDTDGSD ASEGDEVEST TDADVYGEDD

TTEGPRGGVR LYSCDACPHA VFTTHAALLA HAEEHHADLL PDHARLRRIA QKLNPVWNRA

LNARRNTITS WGKKIFHVAA QRDAGESKMQ EAHRARAQLE CVVRRWHDKA RVFIFGSSVA

**Buffers**

**Buffer D** Tris pH 7.5 10 mM, KCl 200 mM, DTT 1 mM, EDTA 0.5 mM MgCl_2_ 3.2 mM

**Buffer 6** Tris pH 7.5 10 mM, KCl 10 mM, DTT 1 mM, MgCl_2_ 3.2 mM

**RNA substrate**

RET1 RNA *in vitro* substrate was designed in accordance with paper I. Aphasizheva *et al* as 24 nt GCUAUGUCUGCUAACUUGUUUUUU and supplied by Dharmacon. (Aphasizheva I, 2004)

**Screening compounds**

The screening set of 3,000 compounds was curated from the NCGC FDA Pharmacology library. Compounds identified for follow up screening were stored as 10 mM or 20 mM stock solution in DMSO.

**Determination of RNA substrate K_m_ for Cid1**

Reactions were carried out in duplicate and contained; 0.1 µl (0.2 units) of Cid1 enzyme (NEB M0337S), UTP at final concentration of 18 uM, (UTP Km determined by Paola Munoz-tello et al, (Munoz-Tello P, 2014)) in a total reaction volume of 10 µl of buffer 6. Using the same substrate as for the RET1 reactions, RNA concentration was varied from 1 nM to 1 µM. Reactions were carried out at 37 °C for 20 mins and then the enzyme heat inactivated at 65 °C for 10 mins. The K_m_ was found to be 3 nM.

**IC50 for Cid1 enzyme**

IC50s were carried out as for RET1, but chemicals stocks were added to a final concentration of 1 mM to 1 µM.

Duplicate reactions were set up with each containing, 0.2 units of Cid1 enzyme, NEB (M0337S) with UTP at final concentration of 18 µM, RNA 3 nM, and buffer 6 up to a final volume of 10 µl. The reaction was done at 37°C for 20 mins, then the enzyme heat inactivated at 65 °C for 10 mins.

**Computational Methodology**

Sequence alignment was carried out with ClustalW2 (Larkin M A, 2007), followed by manual alignment with BioEdit 7.2.5 (<http://www.mbio.ncsu.edu/bioedit/bioedit.html>). For each target, three independent sets of 1,000 candidate models were generated using Modeller 9.14. (Eswar N, 2006) The top 100 energy-ranked models from each set were subsequently clustered using a set of ~20 key binding site and/or catalytic residues, based on pair-wise RMSD, using GROMACS. (Lindahl E, 2001) Cavity analysis was performed using trj_cavity. (Paramo T, 2014) Visual analysis was performed using VMD (<http://www.ks.uiuc.edu/Research/vmd/>).

The initial conformations for each ligand were generated with Corina (Gasteiger J, 1990) and GOLD (Jones G, 1995) version 5.2 was used for performing the docking experiments. Default settings were applied in each docking run and for every protein structure/ligand combination the top-scoring complex conformation according to the ChemPLP (Korb O, 2009) scoring function were retained for further analysis.

**Supplementary Figures**


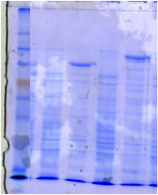

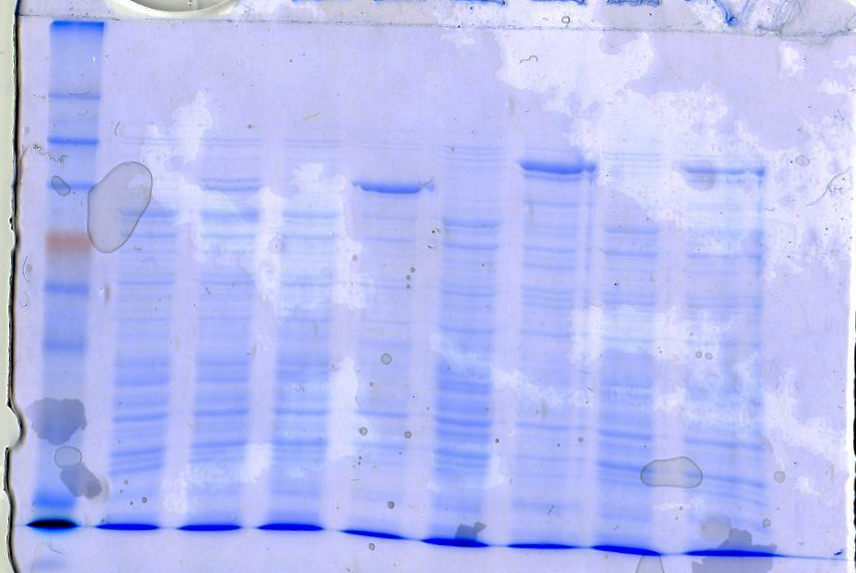


100kDa

150kDa

Induced - +

**Figure S1** GST-RET1 construct 1 production Coomassie stained unpurified un-induced and induced *E. coli* extract.


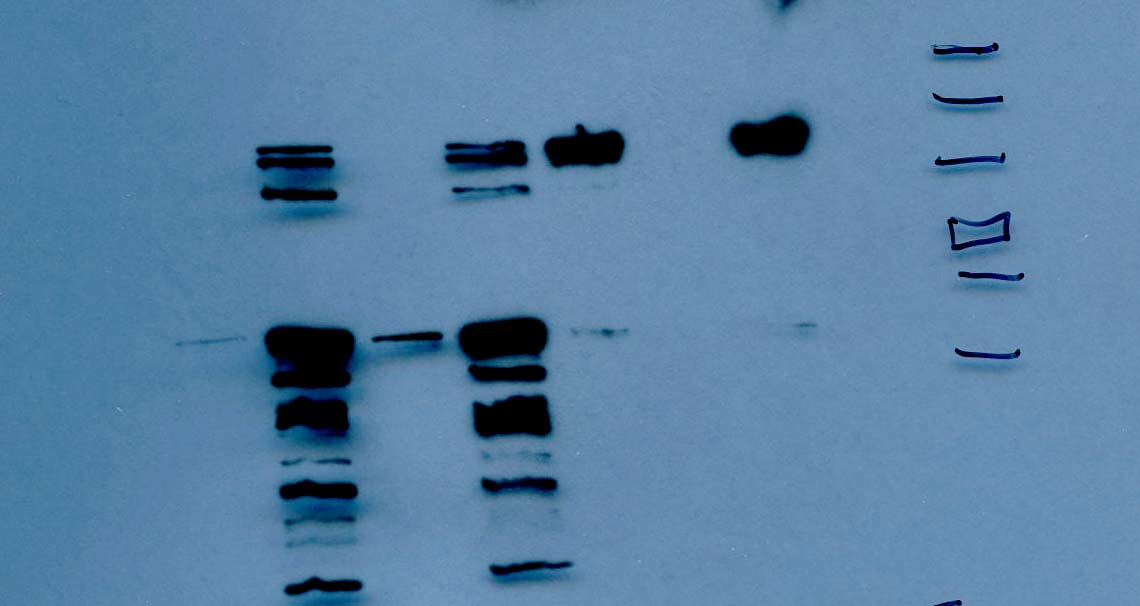


Intact GST-RET1

Inactive GST-RET1 truncation length 463 aa

Purified + + - -

Induced - + + -

**Figure S2** GST-RET1 construct 1 production anti-GST stained after and before purification.
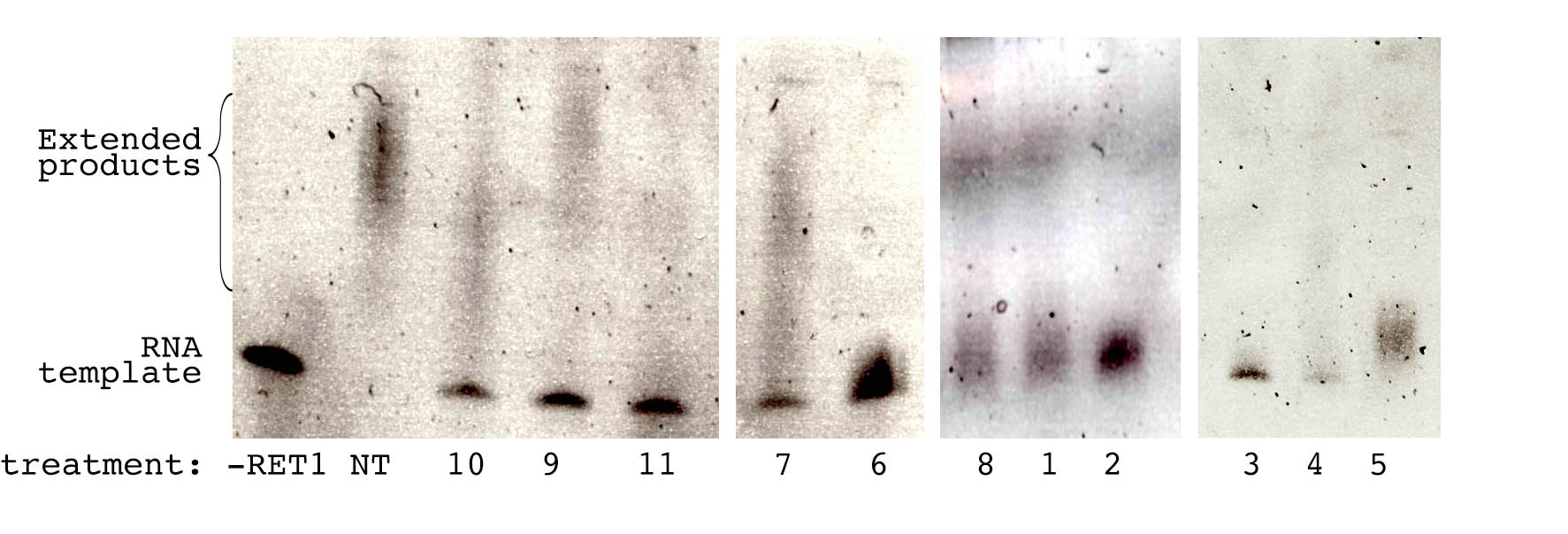


**Figure S3** Orthogonal validation of hit compounds in gel assay shows that RET1 uridylyl transferase activity is inhibited by several compounds identified in the primary screening assay. Lane 1 shows unextended RNA substrate (experiment carried out in the absence of RET1), lane two shows RNA substrate elongated by RET1 (NT = not treated). Subsequent lanes show treatment with RET1 inhibitors 1-11 at 100 μM, and corresponding reduction in RNA uridylylation.

Wt 1% DMSO control

Compound 1, 17 μM

Compound 1, 35 μM

Compound 1, 70 μM

Cell Count (x 10^5^)

Time after addition of Ataciguat (1) (hours)

Cell Count (x 10^5^)

Wt 1% DMSO control

Compound 2, 1.25 μM

Compound 2, 2.5 μM

Compound 2, 5 μM

Time after addition of Exifone (2) (hours)

**Figure S4** Effect of Ataciguat (1) and Exifone (2) on Trypanosome growth.

**Figure S5** Ataciguat (1) docked in RET1 in a horizontally inverted conformation. This conformational cluster, together with the cluster reported in the main text (Fig. 4a) accounts for >95% of total cluster variability.

**Figure S6** Exifone (2) docked in RET1in a horizontally inverted conformation. This conformational cluster, together with the cluster reported in the main text (Fig. 4A) accounts for >95% of total cluster variability.

A

B

**Figure S7** Ataciguat (1) docked in CID1 in two horizontally inverted conformations A) Cluster 1 and B) Cluster 2.

TR|tr|Q8WQX5|Q8WQX5_9TRYP|Q8WQX5_9TRYP MVSKYHRLLQQGLREEEEGVTERNMVAGGEQRHG-HVDDDNAEGDADFYDQKDERRAKMW 59

TR|tr|A4HW38|A4HW38_LEIIN|A4HW38_LEIIN -MSKYSLLFNQGTKDGTDASGS----SGGRTSSSAQTSTTNAS-SPVLLDSAVPSPATAT 54

:*** *::** :: :. . :**. . :.. **. . : *. *.

TR|tr|Q8WQX5|Q8WQX5_9TRYP|Q8WQX5_9TRYP NPKHESANVSAGGKQNRSVRDCLPGSL-------PPVANTST-DAAVRFDRERKNAGHGV 111

TR|tr|A4HW38|A4HW38_LEIIN|A4HW38_LEIIN PPR---------RRLIRRRRGCVGAAEASLSLPEPPQQPQQEEHENVISD----SVHHGS 101

*: : * * *: .: ** . . * * .. **

TR|tr|Q8WQX5|Q8WQX5_9TRYP|Q8WQX5_9TRYP DISCVEGDGAQMGTYVSTGRSDAKAGGGSS---------------------AIGVTADDE 150

TR|tr|A4HW38|A4HW38_LEIIN|A4HW38_LEIIN S----------SGISESDSNVLATSGGGSVTSATPDIAFKVPSPPVASASPSLEGTAALE 151

. * * .. *.:**** :: ** *

TR|tr|Q8WQX5|Q8WQX5_9TRYP|Q8WQX5_9TRYP SDGNLDT-----------DGS-----DASEGDEVESTTDADVYGE---------DDTTEG 185

TR|tr|A4HW38|A4HW38_LEIIN|A4HW38_LEIIN SDGDVVIDDMIKDQEGNSDGSTGATSAAAAGHVMRSDGDSPLSGGGPDPLLPAASCSAAS 211

***:: *** *: *. :.* *: : * . :: .

TR|tr|Q8WQX5|Q8WQX5_9TRYP|Q8WQX5_9TRYP PRGGVRLYSCDACPHAVFTTHAALLAHAEEHHADLLPDHARLRRIAQKLNPVWNRALNAR 245

TR|tr|A4HW38|A4HW38_LEIIN|A4HW38_LEIIN QHIPPRLFTCDMCLHYVSTSYEALEQHALEQHGDALADYTRLRSVAEKLVPVWGEVLQRK 271

: **::** * * * *:: ** ** *:*.* * *::*** :*:** *** ..*: :

TR|tr|Q8WQX5|Q8WQX5_9TRYP|Q8WQX5_9TRYP RNTITSWGKKIFHVAAQRDAGESKMQEAHRARAQLECVVRRWHDKARVFIFGSSVAMGVW 305

TR|tr|A4HW38|A4HW38_LEIIN|A4HW38_LEIIN ASVVQQWGERIFAVAVQRDAGAEKMAEAHRARAQLELVVQRWHPRAKVFIFGSSVAFGVW 331

..: .**::** **.***** .** ********** **:*** :*:*********:***

TR|tr|Q8WQX5|Q8WQX5_9TRYP|Q8WQX5_9TRYP DGTADIDFAVVDVDAMERGSWPPLEKNAVRSITELLRRVGFSFVNLEPISHARVPIIKHH 365

TR|tr|A4HW38|A4HW38_LEIIN|A4HW38_LEIIN DGISDIDFTVVDVEELEAGTWPPSEKNAVRSITELLRRAGFSFINLEPISHARVPIIKHH 391

** :****:****: :* *:*** **************.****:****************

TR|tr|Q8WQX5|Q8WQX5_9TRYP|Q8WQX5_9TRYP ASSPILTVAR-------------------------------------------RDAEDVV 382

TR|tr|A4HW38|A4HW38_LEIIN|A4HW38_LEIIN ASLPIRLTDEQRHRLHEEARQSAAAVDLSTAESLASSSPSSAQEAPAERGHTQLEAELII 451

** ** . . :** ::

TR|tr|Q8WQX5|Q8WQX5_9TRYP|Q8WQX5_9TRYP ARSIRFILNGPATREDRLLLEGSVRDAVGPTGVQQVWWNRTSDMMSATLESTTAAVRAAM 442

TR|tr|A4HW38|A4HW38_LEIIN|A4HW38_LEIIN ARSVRYSLNLPAGPPDSAILEASIRLAVGSAAVQQVWWNRTRDMCCMTFDTTTNAVKAST 511

***:*: ** ** * :**.*:* *** :.********* ** . *:::** **:*:

TR|tr|Q8WQX5|Q8WQX5_9TRYP|Q8WQX5_9TRYP CSPALASASLRTKVQPAHDECRPELYNIDFDLSFRAFGIRNSTLLRKYLLSHPCARPGAI 502

TR|tr|A4HW38|A4HW38_LEIIN|A4HW38_LEIIN CPLHFTSAGMRARVQPLHEECRPELYGMDFDLSFRAFGIRNSHLLRRYLLSHPCARPGAL 571

* ::**.:*::*** *:******* :************** ***:************:

TR|tr|Q8WQX5|Q8WQX5_9TRYP|Q8WQX5_9TRYP VLKDWSKTSGVNNSVNGYFTSYAINIMWIYYLVQKGYVPYVDPL-EIPESLVNYTDFDPR 561

TR|tr|A4HW38|A4HW38_LEIIN|A4HW38_LEIIN VLKDWSKTSGVNNSVNGYLTSYAINIMWIYYLVHRGVIPYVCPARDIPASLRRNVDADPQ 631

******************:**************::* :*** * :** ** . .* **:

TR|tr|Q8WQX5|Q8WQX5_9TRYP|Q8WQX5_9TRYP YTPMIDPEITNTEREELYKAAGDMLVGFFYFYSFEFDWGHNVISLNRPGITTKRMLGWHV 621

TR|tr|A4HW38|A4HW38_LEIIN|A4HW38_LEIIN YAAMVDPAWTPEERAAMEAQAGELLLGFFYYYAFEFDWANHVVSLNRPGVTTKAALGWDV 691

*: *:** * ** : **::*:****:*:*****...*:******:*** ***.*

TR|tr|Q8WQX5|Q8WQX5_9TRYP|Q8WQX5_9TRYP EDVVPVASTSV-----------------------------------SSG----------G 636

TR|tr|A4HW38|A4HW38_LEIIN|A4HW38_LEIIN EDVAQASGPVPHFSVTGWQHQHNLAGAEGQHGDLHGGAAPAASQTRSSAGHEGMIASNAS 751

***. .:. **. .

TR|tr|Q8WQX5|Q8WQX5_9TRYP|Q8WQX5_9TRYP GGSNVKRHPTRYELCIEDPYEENLNLGRHIGVTKSLRVRTELYRGLLSLLKEGETRSCVF 696

TR|tr|A4HW38|A4HW38_LEIIN|A4HW38_LEIIN MSARRSRTTTRYSFCIEDPYEENLNLGRHMGVTKTLRVQTELYRGLLSLLKDDLQHCCVF 811

.:. .* ***.:***************:****:***:************: :.***

TR|tr|Q8WQX5|Q8WQX5_9TRYP|Q8WQX5_9TRYP AAADSSGTP-------AA-----------GGKQSAALPARALFKLMALTTQAISESRRLP 738

TR|tr|A4HW38|A4HW38_LEIIN|A4HW38_LEIIN AASANSAGSTDSNGSTVSGAAEPATAAAKTSAEPTELPVRVLYKLMAVSTRELAMARRRH 871

**: .*. .: . : : **.*.*:****::*: :: :**

TR|tr|Q8WQX5|Q8WQX5_9TRYP|Q8WQX5_9TRYP QSNSDNSGRIANGDNESLTEVGGGHRVEGAGVDPASCAGASLSSFGEPPIGVHEKTLESI 798

TR|tr|A4HW38|A4HW38_LEIIN|A4HW38_LEIIN SAT---------------------------------------VTAGAEFPGVPLSGLEAA 892

.:. : * ** . **:

TR|tr|Q8WQX5|Q8WQX5_9TRYP|Q8WQX5_9TRYP FVEKAPMEFQLVRKVWNWHQLIHRLGYKIHR-GHVMPRREVGVRCTARRDAEETT----- 852

TR|tr|A4HW38|A4HW38_LEIIN|A4HW38_LEIIN FLVQAPTEWKLATQVWNKHQLLHRLGLKLHAREYVLPRREVGVRRLASKAPPGVVLASTP 952

*: :** *::*. :*** ***:**** *:* :*:******** * : ..

TR|tr|Q8WQX5|Q8WQX5_9TRYP|Q8WQX5_9TRYP ----------TE--------------------LASGVDTTKSLRPGRGLTDTMLRDLSRG 882

TR|tr|A4HW38|A4HW38_LEIIN|A4HW38_LEIIN EPTFTAEEAAAAAAEGKRAPSLTEHAPISSAEVTQMNQAFLGALPARRLPEDLLLAMTKG 1012

: ::. :: . *.* * : :* :::*

TR|tr|Q8WQX5|Q8WQX5_9TRYP|Q8WQX5_9TRYP YMTLTPEWVAWSAPWVSQHLRGYSRLTTVRSAVADETPPALATV----PSVVKPPT---- 934

TR|tr|A4HW38|A4HW38_LEIIN|A4HW38_LEIIN YSCLTPSWVAWTKPWAALSAWWTDRLHSPSTPPQSEDPLASGASGQGGASPSLPPGAPRH 1072

* ***.****: **.: .** : : .* * * .: * **

TR|tr|Q8WQX5|Q8WQX5_9TRYP|Q8WQX5_9TRYP ----GEAVMGAMRTTRRNAA----------PARRVELLKLWLWRGISKVTPFKSPR 976

TR|tr|A4HW38|A4HW38_LEIIN|A4HW38_LEIIN ISAVPEKSAGAMHQTRTQLRRHVVAETASVSAGRRALRM--LFR------------ 1114

* ***: ** : * * * *:*

**Figure S8** Uniprot sequence alignment between RET1 in *T. brucei* and *L. infantum* Key active site residues marked in red. Homology in adjacent regions is high.

**Supplementary References**

1. *RNA-editing terminal uridylyl transferase 1: identification of functional domains by mutational analysis.* **Aphasizheva I, Aphasizhev R, Simpson L.** 23, 2004, J. Biol. Chem., Vol. 279, pp. 24123-30.

2. *A critical switch in the enzymatic properties of the Cid1 protein deciphered from its product-bound crystal structure.* **Munoz-Tello P, Gabus C, Thore S.** 5, 2014, Nucl. Acids Res., Vol. 42, pp. 3372-80.

3. *Clustal W and clustal X version 2.0.* **Larkin M A, Blackshields G, Brown N P, Chenna R, McGettigan P A, McWilliam H, Valentin F, Wallace I M, Wilm A, Lopez R, Thompson J D, Gibson T J, Higgins D G.** 21, 2007, Bioinformatics, Vol. 23, pp. 2947-48.

4. **Eswar N, Marti-Renom M. A, Webb B, Madhusudhan M. S, Eramian D, Shen M, Pieper U, Sali A.** Comparative protein structure modeling with MODELLER. *In Current Protocols in Bioinformatics, Supplement 15.* s.l. : John Wiley & Sons, Inc.,, 2006, pp. 15:5.6.1–5.6.30.

5. *GROMACS 3.0: a package for molecular simulation and trajectory analysis.* **Lindahl E, Hess B, Van Der Spoel D.** 8, 2001, J. Mol. Model., Vol. 7, pp. 306-317.

6. *Efficient Characterization of Protein Cavities within Molecular Simulation Trajectories: trj_cavity.* **Paramo T, East A, Garzón D, Ulmschneider M. B, Bond P.J.** 5, 2014, J. Chem. Theory Comput., Vol. 10, pp. 2151-2164.

7. *Automatic generation of 3D-atomic coordinates for organic molecules.* **Gasteiger J, Rudolph C, Sadowski J.** 1990, Tetrahedron Comput. Methodol., Vol. 3, pp. 537-547.

8. **Jones G, Willett P, Glen R. C.** 245, 1995, J. Mol. Biol., pp. 43-53.

9. *Empirical scoring functions for advanced protein-ligand docking with PLANTS.* **Korb O, Stützle T, Exner T. E.** 49, 2009, J. Chem. Inf. Model., pp. 84-96.
